# Clinicians’ Perspectives on the Telehealth Serious Illness Care Program for Older Adults With Myeloid Malignancies: Single-Arm Pilot Study

**DOI:** 10.2196/58503

**Published:** 2024-06-27

**Authors:** Marissa LoCastro, Ying Wang, Tristan Yu, Soroush Mortaz-Hedjri, Jason Mendler, Sally Norton, Rachelle Bernacki, Thomas Carroll, Heidi Klepin, Lucy Wedow, Sean Goonan, Hannah Erdos, Brenda Bagnato, Jane Liesveld, Eric Huselton, Benzi Kluger, Kah Poh Loh

**Affiliations:** 1 School of Medicine and Dentistry University of Rochester Rochester, NY United States; 2 Department of Epidemiology University of Rochester Medical Center Rochester, NY United States; 3 River Campus University of Rochester Rochester, NY United States; 4 Division of Hematology Oncology Department of Medicine James P. Wilmot Cancer Institute Rochester, NY United States; 5 School of Nursing University of Rochester Medical Center Rochester, NY United States; 6 Department of Palliative Care Harvard Medical School Boston, MA United States; 7 Divisions of General Medicine and Palliative Care University of Rochester Medical Center Rochester, NY United States; 8 Section on Hematology and Oncology Department of Internal Medicine Wake Forest School of Medicine Winston-Salem, NC United States; 9 Divisions of Palliative Care and Department of Neurology University of Rochester Medical Center Rochester, NY United States

**Keywords:** serious illness conversations, serious illness conversation, SIC, Serious Illness Care Program, SICP, hematologic malignancy, geriatric oncology, acute myeloid leukemia, AML, myelodysplastic syndrome, MDS, cancer, oncology, oncologist, oncologists, metastases, telemedicine, telehealth, tele-medicine, tele-health

## Abstract

**Background:**

Serious illness conversations may help patients avoid unwanted treatments. We previously piloted the telehealth Serious Illness Care Program (SICP) for older adults with acute myeloid leukemia and myelodysplastic syndrome.

**Objective:**

In this study, we aimed to understand the experience of the telehealth SICP from the clinician’s perspective.

**Methods:**

We studied 10 clinicians who delivered the telehealth SICP to 20 older adults with acute myeloid leukemia or myelodysplastic syndrome. Quantitative outcomes included confidence and acceptability. Confidence was measured using a 22-item survey (range 1-7; a higher score is better). Acceptability was measured using an 11-item survey (5-point Likert scale). Hypothesis testing was performed at α=.10 (2-tailed) due to the pilot nature and small sample size. Clinicians participated in audio-recorded qualitative interviews at the end of the study to discuss their experience.

**Results:**

A total of 8 clinicians completed the confidence measure and 7 clinicians completed the acceptability measure. We found a statistically significant increase in overall confidence (mean increase of 0.5, SD 0.6; *P*=.03). The largest increase in confidence was in helping families with reconciliation and goodbye (mean 1.4, SD 1.5; *P*=.04). The majority of clinicians agreed that the format was simple (6/7, 86%) and easy to use (6/7, 86%). Clinicians felt that the telehealth SICP was effective in understanding their patients’ values about end-of-life care (7/7, 100%). A total of three qualitative themes emerged: (1) the telehealth SICP deepened relationships and renewed trust; (2) each telehealth SICP visit felt unique and personal in a positive way; and (3) uninterrupted, unrushed time optimized the visit experience.

**Conclusions:**

The telehealth SICP increased confidence in having serious illness conversations while deepening patient-clinician relationships.

**Trial Registration:**

ClinicalTrials.gov NCT04745676; https://www.clinicaltrials.gov/study/NCT04745676

## Introduction

Serious illness conversations (SICs) are designed to identify the values, priorities, and concerns of patients with life-threatening illnesses, with the intent of providing them with the information and care that align with their unique needs [1,2]. Explicit elicitation of patient preferences through SICs may prevent unwanted treatments and decrease decisional regret for caregivers during times when patients cannot make decisions themselves [3-6]. SICs are especially important among older adults with hematologic malignancies because they experience poorer outcomes due to aging-related vulnerabilities compared to younger individuals [7-9].

In clinical practice, SICs are often conducted infrequently or late [10,11]. Barriers to SICs reported by clinicians include limited time and clinician-perceived patient discomfort about this topic [10,12]. Additionally, clinicians report that patient and family overestimation of prognosis may cause SICs to remove hope and worsen psychological distress [13,14]. Clinicians are also uncertain about when in the disease course (ie, at the time of diagnosis, after treatment initiation, etc) SICs should occur and which patients would benefit most [11]. The lack of clear guidelines and standardized methods makes it challenging for clinicians to conduct high-quality SICs in clinical practice. The development of interventions and systems-based changes that provide clinicians with standard tools may increase the frequency and improve the quality of SICs for clinicians and their patients.

We previously conducted a pilot study testing the telehealth Serious Illness Care Program (SICP) for older adults with acute myeloid leukemia (AML) and myelodysplastic syndrome (MDS) [15]. The telehealth SICP includes tools and system-based changes to promote early and ongoing SICs [15,16]. The *Serious Illness Care Guide*, one of the telehealth SICP tools, is a script that clinicians are formally trained to use in order to share challenging news related to prognosis and elicit patients’ values, preferences, and concerns regarding their care [15,16]. System-based changes including clinician preparation emails and electronic medical record (EMR) documentation templates provide further support [15,16]. In this analysis, we describe oncology clinicians’ experience with the telehealth SICP.

## Methods

### Study Design and Population

The design and primary outcomes, including patient and caregiver measures, for the single-arm pilot study have previously been published [15]. Oncologists and advance practice providers (APPs) received training to deliver the telehealth SICP to their patients. Enrolled clinicians (oncologists and APPs) who cared for at least 1 older adult with AML or MDS in the past year were eligible.

### Ethical Considerations

This study was approved by the University of Rochester research subjects review board (STUDY00005809). Informed consent was obtained by all study participants, and data were deidentified to safeguard participant information. Participants were not compensated for participation in this research.

### Recruitment

Eligible clinicians were identified by the study team and approached via email. Consented clinicians completed demographics and were scheduled for a 3-hour web-based training session led by a palliative care clinician to learn how to use the *Serious Illness Care Guide*, a component of the telehealth SICP. Once clinicians completed training, the study team identified eligible patients from clinic schedules. Consented patients (and caregivers, if available) and clinicians were scheduled for a telehealth SICP visit either during clinic hours or during admin hours depending on clinician preference. At the end of the study, clinicians completed postintervention measures followed by an audio-recorded interview to discuss their experience delivering the telehealth SICP. Interviews were transcribed by a professional service and uploaded to MAXQDA software (VERBI Software GmBH) for analysis.

### Telehealth SICP

The telehealth SICP has previously been described [15,16]. In brief, it includes preparatory materials, a scheduled telehealth visit, and postvisit materials. Preparatory materials include geriatric assessment results for the patient (provided to the clinician), a patient preparation pamphlet, and a clinician preparation email. The postvisit materials include a family guide for patients and a documentation template for the EMR for clinicians.

### Measures

Baseline measures were collected after the web-based training session and prior to the clinician’s first telehealth SICP visit with a patient. Postintervention measures were collected at the end of the study after the clinician completed all study visits.

### Clinician Confidence

Clinician confidence in having SICs was evaluated at baseline and postintervention time points using the 22-item clinician confidence survey (range 1-7; a higher score indicates more confidence in a given item) [17]. This survey was completed after the web-based training session and at the end of the study.

### Clinician Acceptability

Acceptability of the telehealth SICP was evaluated at the postintervention time point using the 11-item acceptability survey [17]. This survey asked clinicians to rank how helpful the telehealth SICP was in various aspects of care on a scale of 1 to 5 (a higher score is better).

### Statistical Analysis

Descriptive statistics were used to summarize demographics. Paired 2-tailed *t* tests or Wilcoxon 2-sided signed rank tests were used to examine changes from baseline to the postintervention time point, depending on the distribution of data. Due to the small sample size and pilot nature of this study, we performed hypothesis testing at α=.10 (2-tailed). For qualitative analysis of interviews with clinicians, 2 coders (ML and TY) used open coding to identify themes. Thematic saturation was met, and qualitative results were reported according to the COREQ (Consolidated Criteria for Reporting Qualitative Research) guidelines (Multimedia Appendix 1).

## Results

### Overview

A total of 10 clinicians were included in this study (Table 1): 6 oncologists and 4 oncology APPs. The mean age of the clinicians was 42 (SD 12.7) years. The majority of the clinicians were female (6/10, 60%), White (9/10, 90%), and non-Hispanic (10/10, 100%). The mean number of telehealth SICP visits per clinician was 1.9 (SD 1.4; range 0-4). Demographic information for patients was previously published [15]. The mean age of patients was 75 (SD 5.9) years, and the majority were White (17/20, 85%) and non-Hispanic (16/20, 80%). Approximately half (9/20, 45%) were female. Each patient had 1 telehealth SICP visit with their clinician, and a total of 19 (95%) out of the 20 visits took place. The average time per visit was 36.5 (SD 15.4; range 19.3-71.3) minutes.

**Table 1:** Demographics.

Variable			Clinicians (n=10)		Patients (n=20)
**Age (years)**					
	Mean (SD)	42 (12.7)		75 (5.9)	
	Range	26-66		63-87	
**Number of years in practice after completion of training**					
	Mean (SD)	11 (9.8)		N/A^a^	
	Range	2-30		N/A	
**Discipline, n (%)**					
	Oncologist	6 (60)		N/A	
	Advanced practice provider	4 (40)		N/A	
**Sex, n (%)**					
	Male	4 (40)		11 (55)	
	Female	6 (60)		9 (45)	
**Ethnicity, n (%)**					
	Non-Hispanic or Latino	10 (100)		16 (80)	
**Race, n (%)**					
	Asian	1 (10)		0 (0)	
	Black or African American	0 (0)		1 (5)	
	White	9 (90)		17 (85)	
	Unknown or not reported	0 (0)		2 (10)	

^a^N/A: not applicable.

### Clinician Confidence

**Figure 1:**
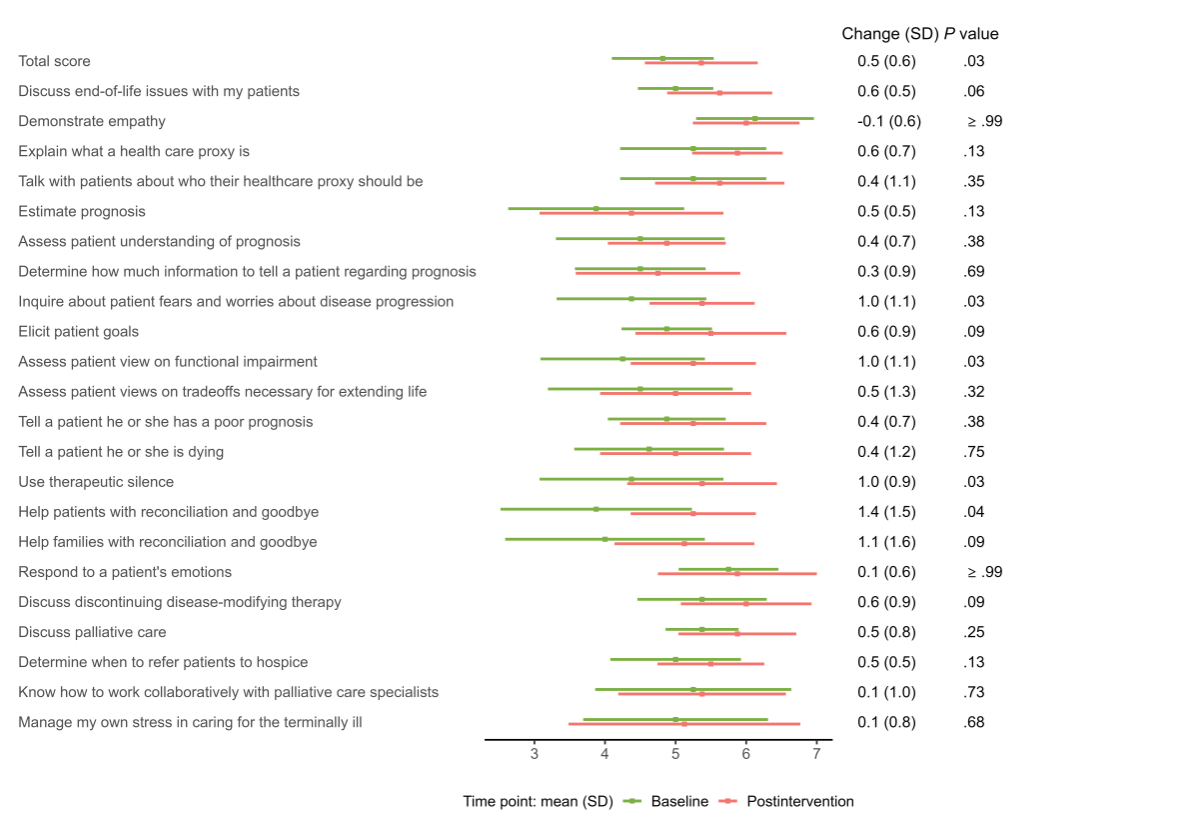
Clinician confidence at baseline and postintervention time points (n=8).

A total of 8 clinicians completed the confidence survey, and 2 clinicians did not complete the confidence measure because they did not enroll any patients. Busy clinical schedules were the major barrier to enrollment for these clinicians. Overall, clinician confidence had a statistically significant increase from baseline to the postintervention time point, with a mean increase of 0.5 (SD 0.6; *P*=.03). At baseline, clinicians were the least confident in helping patients with reconciliation and goodbye (mean 3.9, SD 1.4) and in estimating prognosis (mean 3.9, SD 1.2), and the most confident in demonstrating empathy (mean 6.1, SD 0.8). At the postintervention time point, clinicians were the least confident in estimating prognosis (mean 4.4, SD 1.3) and the most confident in demonstrating empathy (mean 6.0, SD 0.8) and discussing discontinuing disease-modifying therapy (mean 6.0, SD 0.9). From baseline to the postintervention time point, the largest increase in confidence was seen in helping families with reconciliation and goodbye, with a mean increase of 1.4 (SD 1.5; *P*=.04). Additional confidence results are shown in Figure 1.

### Clinician Acceptability

**Figure 2:**
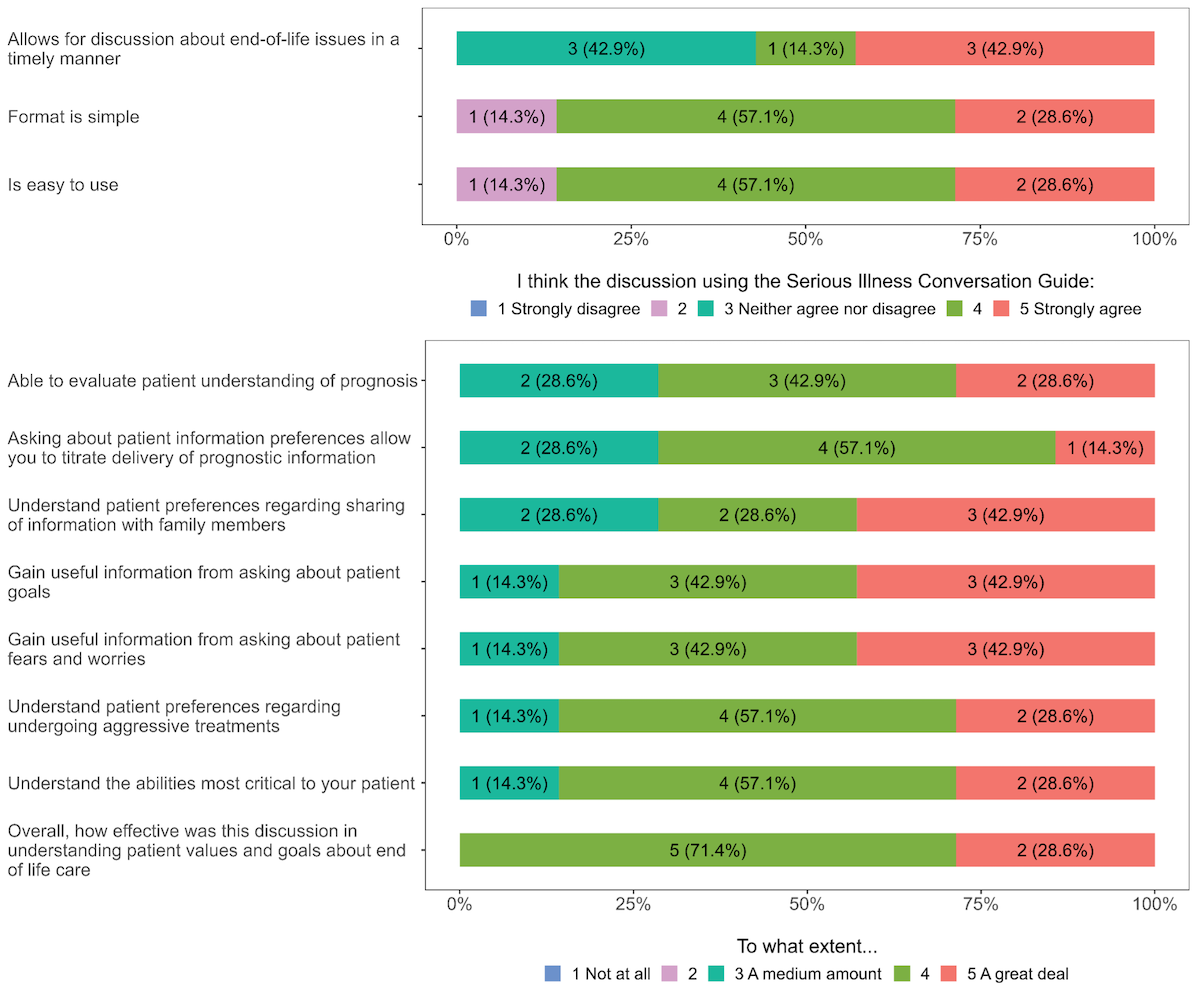
Clinician responses for serious illness care program acceptability (n=7).

A total of 7 clinicians completed the acceptability survey, 2 clinicians did not enroll any patients, and 1 clinician did not complete it. The majority of responding clinicians agreed or strongly agreed that the telehealth SICP format was simple (n=6, 86%) and easy to use (n=6, 86%). Approximately half (n=4, 57%) of clinicians agreed or strongly agreed that the telehealth SICP allowed for timely end-of-life discussions. The majority of clinicians felt that they gained useful information by asking about the patient’s goals (n=6, 86%) and that the telehealth SICP was effective in understanding their patient’s values about end-of-life care (n=7, 100%). Additional acceptability results are shown in Figure 2.

### Qualitative Results

#### Overview

The following qualitative themes emerged (Multimedia Appendix 2): (1) the telehealth SICP deepened relationships and renewed trust; (2) each telehealth SICP visit felt unique and personal in a positive way; and (3) uninterrupted, unrushed time is preferred to optimize the visit experience.

#### Theme 1: The Telehealth SICP Deepened Relationships and Renewed Trust

Clinicians appreciated the opportunity to learn and understand their patients’ preferences, noting that these topics were not routinely addressed during typical clinic visits.

It is meaningful just to spend that time together, and I think hopefully it allows the patient to feel we care about these issues that aren’t just related to specifics of treatment like blood count numbers and things like that. I think just having that space to discuss these more sensitive issues we may not get to at every visit was meaningful.

Clinician #4

Clinicians felt that these conversations were a way to get patients and their families on the same page regarding the patient’s wishes and values for care.

It was good to see real time the amount of support in her support system and how everybody was more or less on the same page.

Clinician #5

Ultimately, these discussions allowed clinicians to renew trust with their patients and their families, deepening their relationship and better preparing all individuals for the future.

I’ve been able to see him since; there’s this renewed trust between us and alliance that I’m going to tell you when things aren’t looking good and I’m going to be transparent with you. Yeah, it was meaningful in both situations to hear the patients say “I really trust you and I’m expecting you’re going to share with me when I need to hear things.”

Clinician #8

#### Theme 2: Each Telehealth SICP Visit Felt Unique and Personal in a Positive Way

Clinicians noted that it is necessary to tailor each telehealth SICP to each patient’s unique needs based on their relationship with the patient and the clinical status of their AML or MDS.

I think there will always be challenges about this—people are at different places in their own journeys with these issues.

Clinician #2

Despite this anticipated challenge, clinicians were surprised at how well each telehealth SICP visit went with their patients.

I thought it would have gone a little worse than I originally expected, because it’s a difficult conversation, through telehealth, but it actually ended up pretty good at the end. I think you just have to do it and don’t feel like the conversation is about taking away hope or end of life. The conversation is really just about what’s important to the patient and the family, and then going in-depth about things you don’t usually talk about. I think you’ll be surprised at the end how good the conversation is and how much it helps you understand the patients and families more.

Clinician #9

Clinicians also appreciated how vulnerable patients were during discussions.

I was happily surprised by how personal it got—both of [the patients] were very open to tell me about their family and their reason for doing what they were doing. Yeah, just being very vulnerable.

Clinician #8

After completing their telehealth SICP discussions, clinicians felt confident and ready to have additional visits with other patients.

Getting over my discomfort before the first one, because it was the first time I’d ever done a script with a patient. There was that, and I was also a little bit apprehensive about addressing the broader aspects of their care. I think after I started that first one and got through it and realized how good it felt like it went from both the patient’s standpoint and mine, I felt like the other ones were really smooth.

Clinician #1

#### Theme 3: Uninterrupted, Unrushed Time Is Preferred to Optimize the Visit Experience

Clinicians noted that it is challenging to know when the right time is to have a telehealth SICP visit with a given patient.

I think the key challenge has just been finding the time to be thoughtful about “Who can I have this discussion with?” in a programmatic way when it’s just so busy where I often don’t even know what patient I’m seeing until they’re in the room.

Clinician #10

Additionally, clinicians emphasized that it is important for telehealth SICP visits to be uninterrupted so that they can provide their full, undivided attention to the conversation.

On clinic days…most of your patients might be getting treatments too, that you’re getting more interruptions as far as phone calls or from nursing in clinic. Whereas on…a continuity day…I can at least try to be unbothered for an hour. Not that I won’t get paged, but…I seem to get less interruptions on the continuity day than a clinic day.

Clinician #6

Despite these challenges, clinicians highlighted how important these visits are for patients and suggested scheduling them outside of clinic time to maximize time and ensure visits are uninterrupted.

Yeah. I think you just have to schedule—it has to be its own independent meeting. I think it just works best…for the patients to sort of come knowing that’s the conversation they’re going to have versus integrate it into a regular clinic visit based on what’s happening on that clinic day.

Clinician #3

For visits taking place during clinic time, clinicians felt that using telehealth is a good option to make SICP conversations feel different than a typical clinic visit.

I do like the telehealth aspect of it just because I feel like when you’re in clinic for better or worse you are in the clinic mindset of trying to get through your day and certainly for new patients or patients experiencing change, there’s time to readdress these goals of care but in the middle of a busy clinic day for a patient who’s stable on therapy, often these conversations don’t come up, so having that separate time in clinic is important. I think patients really like the telehealth visit for a variety of reasons, so I think that’s helpful for the patients.”

Clinician #4

## Discussion

### Principal Findings

We found that the telehealth SICP increased clinician confidence in having SICs, and clinicians found it was a simple and easy way to understand patient values about their care. Furthermore, these conversations deepened patient-clinician relationships and renewed trust.

### Comparison With Prior Work

Previous literature has noted psychological distress for both patients and clinicians as a concern of SICs [10,18]. Nevertheless, we found that these discussions enhanced and improved the patient-clinician relationship. We previously published that patients in this study experienced no change in anxiety, depression, or distress between baseline and postintervention time points, further underscoring that SICs are not detrimental to emotional well-being [15]. Previous studies have also shown that SICs do not take away hope from patients [17,19]. In fact, patients have reported increased control over their illness following SICs [15,20]. Similarly, after SICs, clinicians have reported improved satisfaction with their role in patient care leading to decreased clinician anxiety and distress [21-23]. Moral distress, especially around end-of-life planning, has been cited as a major cause of burnout for oncology clinicians [22,23]. SICs, therefore, have the potential to combat clinician burnout while decreasing anxiety and distress for patients and clinicians.

We found that clinicians reported low levels of confidence in estimating prognosis before and after telehealth SICP visits. Estimating prognosis is complex; nonetheless, prognosis remains at the forefront of conversations for many older adults with cancer [24-26], and there are a variety of standardized tools, such as European Leukemia Net risk stratification for AML and Revised International Prognostic Scoring System for MDS, to help estimate patients’ prognosis [27-29]. This is of vital importance because prognosis is necessary for clinicians to offer effective treatment recommendations and for patients and their loved ones to make advanced care planning decisions [30,31]. Indeed, most of the available data strongly suggest that patients with cancer want to receive prognostic information from their clinicians, and therefore prognosis needs to be effectively addressed through SICs [16,32]. It is therefore vital that clinicians work to become comfortable with, and skilled at, sharing uncertainty around prognosis [33,34]. Doing so has been shown to increase patient engagement and satisfaction [33,34].

Clinicians felt that uninterrupted, unrushed time is necessary to optimize visits, but time limitations have frequently been cited as a barrier to SICs in clinical practice [10,35]. One way to increase time and efficiency is to provide note templates for SIC documentation, similar to the EMR documentation template provided to clinicians in this study. A previous study found that the use of a note template saved clinicians 23 minutes per patient [36]. Note templates may also allow clinicians to clearly and efficiently identify the patient’s values in subsequent medical encounters [37,38]. A second way to optimize time is the use of telehealth, which enhances clinic efficiency for clinicians, leading to reduced wait time for patients [39-41]. Telehealth further benefits patients by increasing convenience and decreasing the burden through eliminating the need for travel [16,39,40]. The telehealth SICP, which includes both of these solutions, is one way to optimize time for high-quality SICs.

### Limitations

The strengths of this study include that we used both quantitative and qualitative analyses to thoroughly assess clinicians’ experience with the telehealth SICP. Additionally, our intervention aimed to support a vulnerable and underrepresented group of patients—older adults with hematologic malignancies. The limitations of this study include that it was a single-center design with a small sample size and limited diversity of clinicians and patients. Additional research to understand the experience of the telehealth SICP in underrepresented populations is warranted [42]. We were able to include only 1 community clinician, and even so, it was only academic oncologists who ultimately enrolled patients.

### Conclusions

The telehealth SICP has the potential to help clinicians understand what matters most to patients by improving their confidence in having SICs. Clinicians experienced unique SICs with each patient and felt an overall renewed sense of trust and partnership with patients and their families. This work highlights the potential for SICs to lead to more collaborative and patient-centered care both up-front and at end-of-life for older adults with AML and MDS. We plan to continue to explore the efficacy of the telehealth SICP in a future randomized controlled trial, as well as its implementation strategies.

## Appendix

Multimedia Appendix 1 COREQ (Consolidated Criteria for Reporting Qualitative Research) guidelines for qualitative analysis.

Multimedia Appendix 2 Additional exemplar quotes.

## Abbreviations

AML: acute myeloid leukemia
APP: advance practice provider
COREQ: Consolidated Criteria for Reporting Qualitative Research
EMR: electronic medical record
MDS: myelodysplastic syndrome
SIC: serious illness conversation
SICP: Serious Illness Care Program
