# Supplementary material for: Clinicians’ Perspectives on the Telehealth Serious Illness Care Program for Older Adults With Myeloid Malignancies: Single-Arm Pilot Study
Source: JMIR Form Res. 2024 Jun 27;8:e58503. doi: 10.2196/58503 (PMC11240066; doi:10.2196/58503)
Supplement: Multimedia Appendix 1 [file formative_v8i1e58503_app1.docx]

| No. | Item | Guide questions/description |
| --- | --- | --- |
| Domain 1: Research team and reflexivity | | |
| 1. | Interviewer/facilitator | Marissa LoCastro (ML): medical student |
| 2. | Credentials | ML: BS |
| 3. | Occupation | ML: medical student |
| 4. | Gender | Female |
| 5. | Experience and training | Trained by KPL (MD); prior experience interviewing patients, caregivers, and clinicians (advanced practitioners, physicians, nurses). |
| 6. | Relationship established | Interviewer/interviewee relationship started at recruitment and ended at the end of study. |
| 7. | Participant knowledge of the interviewer | Participants understood the interview was intended to elicit feedback and understand their experience delivering the telehealth Serious Illness Care Program (SICP). |
| 8. | Interviewer characteristics | The interviewer did not report personal information that may lead to bias. |
| Domain 2: Study design | | |
| 9. | Methodological orientation and theory | The aim of this study was to understand clinician’s experience delivering the telehealth SICP to their patient. |
| 10. | Sampling | An eligibility criterion was pre-defined. Eligible clinicians cared for at least 1 older adult with acute myeloid leukemia or myelodysplastic syndromes at the Wilmot Cancer Institute in the last year. |
| 11. | Method of approach | Eligible patients were identified by the study team by screening clinicians staffed at Wilmot Cancer Institute. Eligibility was confirmed with the principal investigator. Once eligibility was confirmed, the study team approached clinicians via email. |
| 12. | Sample size | 10 participants |
| 13. | Non-participation | All approached clinicians consented to the participate. |
| 14. | Setting of data collection | Interviews were conducted over zoom or via telephone. |
| 15. | Presence of non-participants | There were no non-participants present at interviews. |
| 16. | Description of sample | Demographics are described in Table 1. |
| 17. | Interview guide | An interview script was used by the interviewer for semi-structured interviews. |
| 18. | Repeat interviews | No repeat interviews were conducted. |
| 19. | Audio/visual recording | With the permission of participants, interviews audio-recorded interviews and professionally transcribed. |
| 20. | Field notes | Field notes were not taken. |
| 21. | Duration | Interviews lasted approximately 15-30 minutes. |
| 22. | Data saturation | Data and thematic saturation were achieved for this study. |
| 23. | Transcripts returned | Participants did not receive returned transcripts. |
| Domain 3: analysis and findings | | |
| 24. | Number of data coders | Two coders (ML and TY) |
| 25. | Description of the coding tree | An initial coding schema was developed and subsequently adapted to fit themes emerging from the data. |
| 26. | Derivation of themes | Themes were developed using open coding and focused content analysis. |
| 27. | Software | MAXQDA (VERBI Software GmbH, Berlin, Germany) |
| 28. | Participant checking | Participants were not consulted to provide feedback on themes. |
| 29. | Quotations presented | Quotations are available in the Results section and Supplemental Table 2. |
| 30. | Data and findings consistent | ML and TY met to discuss and confirm findings. |
| 31. | Clarity of major themes | Major themes are described in the Results and Supplemental Table 2. |
| 32. | Clarity of minor themes | Minor themes were not described in this analysis. |
