# Supplementary material for: Clinicians’ Perspectives on the Telehealth Serious Illness Care Program for Older Adults With Myeloid Malignancies: Single-Arm Pilot Study
Source: JMIR Form Res. 2024 Jun 27;8:e58503. doi: 10.2196/58503 (PMC11240066; doi:10.2196/58503)
Supplement: Multimedia Appendix 2 [file formative_v8i1e58503_app2.docx]

| Theme | Exemplar Quotes |
| --- | --- |
| The telehealth SICP deepened relationships and renewed trust. | Clinician 9: *“We got everybody on the same page with the family members involved, but the patient was able to verbalize what was important to him…A lot of the talking was done by the family members…but [the patient] was able to express what was important to him and after the visit, I felt like they were able to talk more within themselves.”*  Clinician 1: *“They all deepened my relationships with those patients. I think they made me feel better that – all of the patients were in a difficult spot with their disease so it made me feel good that we were really trying to address the end of their life. I just felt like for all of them I got to know them better. I felt like there was really no downside to it and it was just really helpful getting to know the patients better, deepening the relationship and really helping plan the end of life and what they would want at end of life. There were a lot of positives.”*  Clinician 6: *“As you know, two of [the patients] passed away I definitely think it was a positive thing because I think it brought us closer too and made, at the end, having to say goodbye easier, too.”* |
| Each telehealth SICP visit felt unique and personal in a positive way. | Clinician 5: “*Each of the visits I thought were very different from each other, partly because with one of the patients, we have known each other for many, many years. Because of that, I felt it was harder to stay on task. With [the other patient], I felt it was the best of the 3 of them. I was able to follow the script to a T without sounding scripted and I felt like the conversation flowed well and that I learned a lot from [the patient] about what’s important to her. I learned a lot from [the patient I’ve known for a long time] too, but I already kind of knew where she was at.”* |
| Uninterrupted, unrushed time is preferred to optimize the visit experience. | Clinician 8: “*The one obstacle I felt with this trial was timing of everything…In terms of addressing when we should be having these conversations with patients, I think it’s really hard to say and I think it’s really patient specific. There are patients you’ve probably brought up to me before that now would feel appropriate to have those conversations with, but we need our footing. Then there are other patients where I think we should be having this conversation day 1 because they need to understand what’s coming.”*  Clinician 5: *“I could see [doing telehealth SICP visits during clinic] having a downstream effect if it went over – and [one patient] was much longer than the allotted time, but it was good because I didn’t have anybody after her. So I think it might make sense in the future to do them on off days.”* |
